# Idiopathic Hypertrophic Pachymeningitis with Elevated Anti-Thyroglobulin Antibodies—A Case Report

**DOI:** 10.3390/reports9010015

**Published:** 2025-12-31

**Authors:** Paweł Pobudejski, Mateusz Toś, Katarzyna Zawiślak-Fornagiel, Joanna Siuda

**Affiliations:** Department of Neurology, Faculty of Medical Sciences in Katowice, Medical University of Silesia, Medykow 14 Street, 40-752 Katowice, Polandjsiuda@sum.edu.pl (J.S.)

**Keywords:** idiopathic hypertrophic pachymeningitis, pachymeningitis, thyroid autoimmunity, anti-thyroglobulin antibodies, case report

## Abstract

**Background and clinical significance**: Idiopathic hypertrophic pachymeningitis (IHPM) is a rare inflammatory disorder characterized by diffuse or focal dural thickening and heterogeneous presentations. We report a corticosteroid-responsive IHPM with elevated anti-thyroglobulin (anti-Tg) antibodies despite oncologic control after thyroidectomy. This case suggests that systematic assessment for autoimmunity should be a standard component of the IHPM work-up. **Case presentation**: A 77-year-old woman presented with recurrent vertigo, imbalance, and headaches. Brain MRI showed diffuse pachymeningeal thickening with mild heterogeneous enhancement, radiologically stable over >2 years. Extensive evaluation excluded infectious, neoplastic (including paraneoplastic), cerebrospinal fluid hypotension and systemic autoimmune causes; findings did not support IgG4-related disease. Thyroid work-up revealed hypothyroidism with multinodular goiter; total thyroidectomy was performed, and there was no indication for adjuvant radioiodine therapy. Despite oncologic control, anti-Tg antibodies remained markedly elevated, while anti-thyroid peroxidase antibodies (anti-TPO) declined. Symptoms repeatedly improved with oral methylprednisolone and recurred on taper; adverse effects were mild and manageable. The patient remains under clinical and oncologic surveillance with symptom-guided steroid re-challenge. **Conclusions**: IHPM may exhibit a dissociation between clinical response and radiologic course. Persistently elevated anti-Tg after thyroidectomy can coexist with IHPM and may signal ongoing autoimmunity rather than active cancer.

## 1. Introduction and Clinical Significance

Idiopathic hypertrophic pachymeningitis (IHPM) is a rare disease characterized by diffuse or localized thickening of the dura mater, which may or may not be accompanied by localized fibrosing inflammation [1,2]. The thickening can be located within the cranial or spinal dura mater, less commonly in both locations simultaneously [3,4].

Reports suggest that men are affected more frequently than women, and the disease typically manifests in the sixth or seventh decade of life [1,3]. However, there are also descriptions in the literature of pediatric patients as young as 3 years old, presenting with radiologically detected abnormalities and accompanying clinical symptoms [2,5,6].

The pathogenesis of thickening of the dura mater—hypertrophic pachymeningitis—is heterogeneous and may include traumatic, infectious, neoplastic or systemic inflammatory causes (Table 1). When no underlying disease is identified, the condition is referred to as IHPM, as a diagnosis of exclusion.

IHPM in its spinal form was first described by Charcot and Hoffrey in 1869 [3,4], and the cranial form was first reported by Naffziger and Stern in 1949 [3]. The spectrum of clinical manifestations is broad. In most patients, the first and predominant symptom is headache, usually chronic and of varying intensity. Other possible manifestations include cranial neuropathies—particularly of the optic, oculomotor, facial or vestibulocochlear nerves—as well as focal neurological deficits, cerebellar ataxia and signs of increased intracranial pressure [1,8,12].

The aim of this report is to present a case of chronic pachymeningitis in a patient with persistently elevated anti-thyroglobulin antibody (anti-Tg) levels, whose symptoms responded to corticosteroid therapy despite stable neuroimaging findings.

## 2. Case Presentation

A 77-year-old woman with a medical history of arterial hypertension, irritable bowel syndrome, osteoarthritis and gastroesophageal reflux disease was admitted to the emergency department in January 2022 due to vertiginous symptoms that worsened in the supine position, did not improve with eye closure and were accompanied by nausea and vomiting. Neurological examination revealed only a rapidly fatigable fine nystagmus without other focal deficits. A non-contrast head computed tomography (CT) scan showed no acute focal abnormalities. Because of transient symptom improvement, no further diagnostic work-up was undertaken during hospitalization.

**Figure 1:**
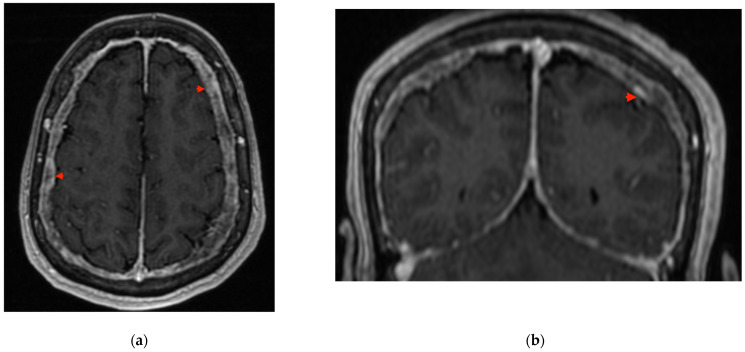
T1-weighted contrast-enhanced MRI images demonstrate dural thickening. The examination was performed in July 2022. (**a**) Sagittal view. (**b**) Coronal view showing the “Eiffel-by-night” sign. Red arrows indicate abnormal dural thickening.

**Figure 2:**
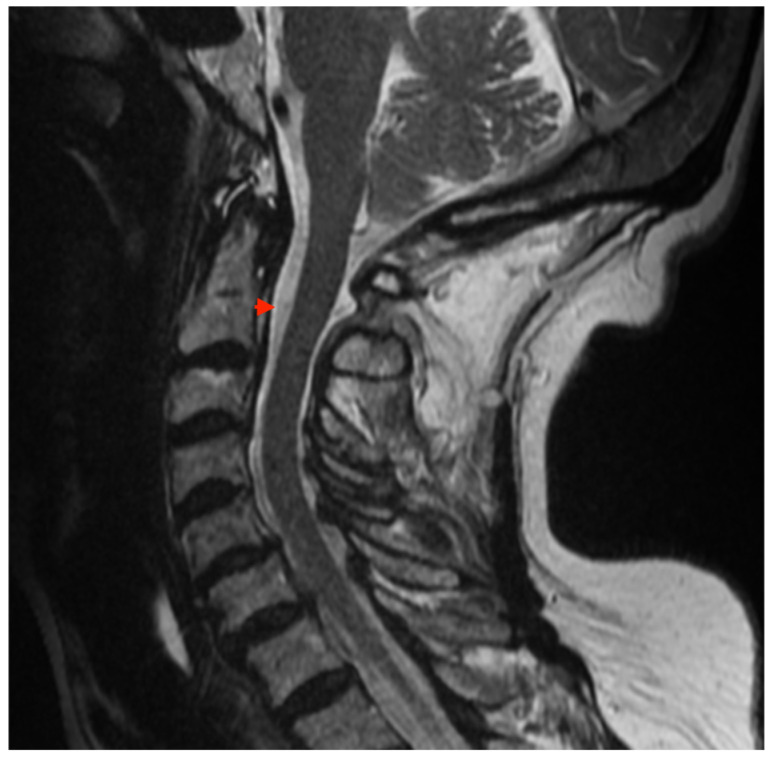
Sagittal T1-weighted contrast-enhanced MRI images of the cervical spine showing suspected thickening of the dura mater. The study was conducted in August 2022. Red arrow indicate abnormal dural thickening.

Due to recurrent episodes of vertigo associated with sudden changes in body position and brief binocular diplopia, further outpatient diagnostic work-up was performed between July and August 2022. Contrast-enhanced brain magnetic-resonance imaging (MRI) revealed diffuse thickening of the dura mater up to 9 mm with slightly nodular contours and pathological, mildly heterogeneous enhancement after intravenous contrast administration (Figure 1). Additionally, the cerebellar tonsils were found to extend approximately 4 mm below the McRae line into the spinal canal. Cervical spine MRI without contrast suggested a small degree of dural thickening in the upper cervical region (Figure 2).

Given these neuroimaging findings, the patient was admitted to the Neurology Department in September 2022 for further differential diagnostic evaluation. On a neurological examination, she presented with an unsteady Romberg test, tending to fall backward and to the left. She also reported a weight loss of approximately 3–5 kg over the preceding several months.

A comprehensive in-hospital work-up was performed, including diagnostic tests for the following:

- Inflammatory and autoimmune diseases: systemic connective tissue disorders, immune-related coagulopathies (including rheumatoid factor (RF), lupus anticoagulant, anti-cyclic citrullinated peptide antibodies (anti-CCP), antinuclear antibodies (ANA), anti-double-stranded DNA (anti-dsDNA), COMBI panel—AMA, ASMA, LKM, and anti-neutrophil cytoplasmic antibodies (pANCA, cANCA);
- Metabolic and deficiency disorders: calcium-phosphate metabolism, alkaline phosphatase, vitamin D, vitamin B12, folic acid, kappa and lambda light chains;
- Infectious diseases: viral, bacterial and neuroinfectious causes: including testing for syphilis, tuberculosis, hepatitis B and C, Human Immunodeficiency Virus (HIV) and Lyme disease antibodies;
- Neoplastic and paraneoplastic screening: tumor markers (AFP, CEA, CA19-9, ROMA test) and onconeural antibodies (anti-amphiphysin, anti-CV2, anti-PNMA2, anti-Ri, anti-Yo, anti-Hu, anti-recoverin, anti-SOX1, anti-titin, anti-Zic4, anti-GAD65, anti-Tr);
- Endocrine disorders, including thyroid disorders: thyroid function tests (thyroid-stimulating hormone (TSH), free triiodothyronine (fT3), free thyroxine (fT4), thyrotropin receptor antibodies (TRAb), anti-thyroglobulin (anti-Tg) and anti-thyroid peroxidase antibodies (anti-TPO).

Among the numerous diagnostic tests performed, the main abnormalities were the presence of anti-TPO antibodies at a level of 451 IU/mL (local laboratory reference range < 34 IU/mL) and anti-Tg antibodies at a titer of 748.31 IU/mL (local laboratory reference range < 115 IU/mL). The total IgG concentration was within normal limits (1003 mg/dL—reference 700–1600 mg/dL).

A month later, during a follow-up visit at the neurology outpatient clinic, the patient reported persistent vertigo and ongoing weight loss (7 kg over approximately 9 months). A contrast-enhanced chest CT revealed an irregular 7 mm opacity in the IV segment of the right lung, considered most likely non-neoplastic. Due to elevated light chain levels (κ 29.47 mg/L, reference 3.30–19.40 mg/L; λ 12.83 mg/L, reference 5.71–26.3 mg/L), hematologic and internal medicine consultations were recommended, revealing no significant pathology.

Symptomatic treatment with oral methylprednisolone was initiated at an initial dose of 64 mg/day (1.03 mg/kg) with gradual tapering (dosing details presented in Table 2). At a 3 month follow-up, the patient reported complete resolution of vertigo and reduction in balance disturbances; therefore, treatment was continued at 8 mg/day. During further observation, the patient reported intermittent sleep disturbances with frequent awakenings, low mood, and mild transient psychotic symptoms, which did not require discontinuation of corticosteroid therapy.

**Figure 3:**
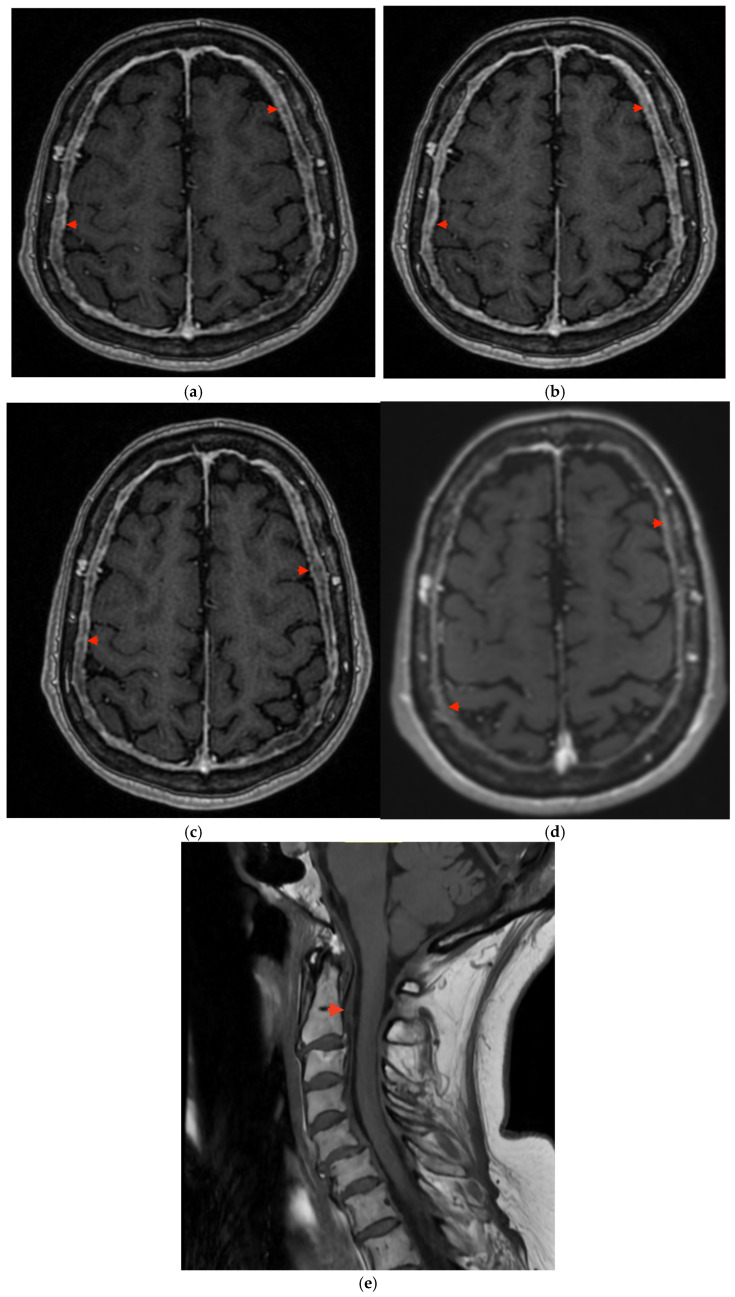
Axial MRI images showing thickening of the meninges obtained in (**a**) April 2023, (**b**) August 2023, (**c**) January 2024, and (**d**) September 2024. (**e**) Suspected thickening of the cervical spinal dura observed in September 2023 (T1). Red arrows indicate abnormal dural thickening.

In the seventh month of corticosteroid therapy, a follow-up contrast-enhanced brain MRI was performed, showing neither progression nor regression of the previously described dural thickening (Figure 3). Consequently, in June 2023, an attempt was made to taper the steroid dose to 4 mg/day. The patient reported worsening vertigo; therefore another neuroimaging follow-up along with symptomatic treatment with flunarizine was recommended and an increase in the methylprednisolone dose to 8 mg/day considered but not advised. A subsequent contrast-enhanced brain MRI performed four months later revealed findings comparable to the previous scans, while a non-contrast cervical spine MRI showed no evident dural thickening (Figure 3).

During the diagnostic process, thyroid hormone and TSH levels were measured. As hypothyroidism was detected, a neck ultrasound with thyroid assessment was performed, revealing a multinodular goiter that required further evaluation. After endocrinological consultation, the patient was referred to the Department of General, Endocrine and Oncological Surgery for surgical management of a substernal multinodular goiter, without preoperative suspicion of malignancy. In December 2023, the patient underwent total thyroidectomy with histopathological sampling. Histopathological examination revealed the follicular variant of papillary thyroid carcinoma (microcarcinoma papillare), classified as pT1a NX, LVI-0, with a 1 mm encapsulated tumor focus located in the right lobe showing involvement of the intrathyroidal capsule without extrathyroidal extension or vascular invasion. Given the early stage of malignancy, the patient was not qualified for adjuvant radioiodine therapy. Post-thyroidectomy oncologic follow-up included serial neck ultrasound examinations, whole-body iodine-131 scintigraphy, and regular clinical follow-up by endocrinology and oncology specialists. These assessments revealed no imaging or clinical evidence of local recurrence or distant metastatic disease during follow-up.

At that time, the patient reported partial improvement of symptoms, including complete resolution of headaches and persistence of mild vertigo, balance disturbances, sleep difficulties (mainly insomnia) and occasional anxiety. Neurological examination revealed mild left limbs ataxia, asymmetry of deep tendon reflexes and an unsteady Romberg test. A gradual reduction in methylprednisolone from 4 mg to 2 mg/day was initiated and flunarizine was discontinued. During subsequent follow-up visits after the initial hospitalization, no limb coordination deficits were observed. The patient reported sustained remission with self-discontinuation of corticosteroid therapy after resolution of symptoms. Subclass analysis demonstrated reduced IgG1 (3.76 g/L—reference 4.05–10.11 g/L) and IgG2 (1.40 g/L—reference 1.69–7.86 g/L) levels. Despite stable radiological findings on a contrast-enhanced brain MRI in January 2024 (Figure 3), a paraneoplastic background of the disease was still considered. At that time, the patient remained under oncological supervision. Follow-up tests included periodic measurements of TSH and serum thyroglobulin levels, as well as neck ultrasound, all of which were within normal limits. However, two consecutive anti-Tg measurements revealed elevated titers of 1130 IU/mL and 1000 IU/mL (reference < 115 IU/mL).

After six months, the patient experienced recurrence of symptoms, including vertigo, headaches, nausea, and vomiting. Methylprednisolone was reintroduced at a dose of 16 mg/day with gradual tapering, which resulted in good clinical improvement. Anti-Tg antibody levels remained elevated but stable across serial measurements.

On subsequent contrast-enhanced brain MRI, dural thickening remained radiologically stable compared with previous scans (Figure 3). Additionally, a small calcified meningioma, retrospectively visible in previous scans, was identified without signs of progression. At the neurological follow-up in October 2024, the patient reported persistent positional vertigo despite ongoing methylprednisolone therapy at a dose of 8 mg/day (Table 3).

During further evaluation, the patient underwent cardiologic consultation and was hospitalized to exclude oncologic progression—the anti-Tg levels were 975 IU/mL (reference < 115 IU/mL). In November 2024, a whole-body iodine-131 scintigraphy performed at the Department of Nuclear Medicine and Endocrinology revealed only minimal tracer uptake in the right thyroid bed, with no abnormal accumulation elsewhere.

Because of persistent symptoms, a CT angiography of cerebral arteries was performed three months later, revealing no vascular abnormalities. During the subsequent neurological visit, the methylprednisolone dose was increased to 16 mg/day. Laboratory tests revealed anti-Tg antibody levels of 1022 IU/mL (reference < 115 IU/mL) and anti-TPO of 78.6 IU/mL (reference < 34 IU/mL), with no other abnormalities suggesting an alternative etiology for the dural thickening. The diagnostic work-up was expanded to include a contrast-enhanced CT scan of the abdomen and pelvis, which revealed multiple hepatic cysts, a right renal cyst, and a small pancreatic cyst.

Due to persistent symptoms, in May 2025 the patient was hospitalized in the Neurology Department for further evaluation and extension of the diagnostic work-up aimed at identifying the underlying cause of the observed abnormalities.

Repeated laboratory assessments revealed a decline in anti-TPO antibody levels (69.7 IU/mL; reference < 34 IU/mL) but persistent elevation of anti-Tg antibodies (1159 IU/mL; reference < 115 IU/mL) (Table 4). Extended endocrine evaluation revealed elevated fasting adrenocorticotropic hormone (ACTH) and prolactin levels, decreased estradiol levels, and increased beta-2 globulin concentrations in cerebrospinal fluid electrophoresis. Other investigations, including cerebrospinal fluid (CSF) analysis, IgG subclasses analysis and electroencephalography (EEG), showed no new abnormalities.

**Figure 4:**
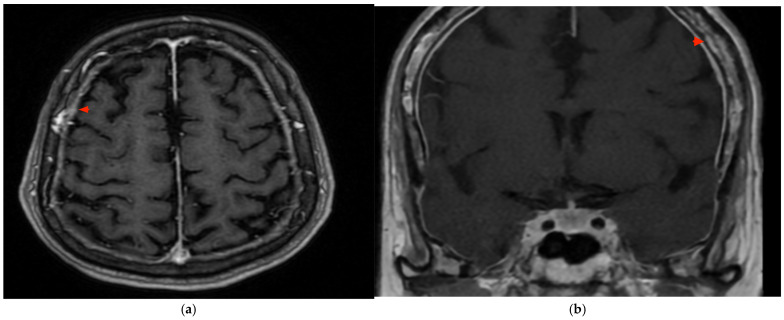
(**a**) Contrast-enhanced brain MRI showing hypertrophic meningitis comparable to previous examinations (**b**). MRI of the pituitary gland demonstrating no thickening of the meninges in the examined region but a subtle bulging of the superior part of the pituitary gland above the sella turcica. Red arrows indicate abnormal dural thickening.

Contrast-enhanced brain MRI demonstrated findings comparable to previous examinations (Figure 4a). MRI of the pituitary gland revealed no thickening of the meninges in the examined region (Figure 4b) but showed a subtle bulging of the superior part of the pituitary gland above the sella turcica.

Three months later, during a follow-up visit, the patient reported a recurrence of symptoms. Neurological examination revealed mild signs of upper limb ataxia (left greater than right), unsteady gait, and a positive Romberg test. Methylprednisolone therapy was reintroduced at an initial dose of 16 mg/day with gradual tapering. After two months the patient reported partial improvement, but persistent symptoms of mild severity. Steroid therapy was deferred due to the mild nature of the symptoms and the stable clinical course. A follow-up evaluation in 6 months and an MRI of the brain in 12 months were recommended.

## 3. Discussion

Given the heterogeneous pathogenesis of dural hypertrophy—confirmed in the presented patient by multiple MRI studies—a broad spectrum of diseases should be considered during diagnostic evaluation, including infectious, neoplastic and autoimmune disorders, in order to exclude secondary causes of the condition. Only after such causes have been ruled out can a diagnosis of IHPM be established as a diagnosis of exclusion.

### 3.1. Differential Diagnosis

#### 3.1.1. Autoimmune Diseases

Scientific evidence suggests that immunologic mechanisms are among the most common causes of hypertrophic pachymeningitis, although some reports point to the presence of an idiopathic component [13]. Therefore, systemic diseases were initially considered in the diagnostic workup, including sarcoidosis, granulomatosis with polyangiitis, Sjögren’s syndrome, rheumatoid arthritis and systemic lupus erythematosus. Accordingly, in September 2022 and May 2025, laboratory tests were performed, including the assessment of ACE (which may be elevated in sarcoidosis), rheumatoid factor (RF), anti-CCP, ANA profile, AMA antibodies, ASMA antibodies, LKM antibodies, anti-neutrophil cytoplasmic antibodies including pANCA, cANCA and anti-dsDNA antibodies, all of which are used in the evaluation of autoimmune diseases. None of the tests revealed serological abnormalities. Throughout the course of the disease, no systemic manifestations—commonly observed in secondary forms of pachymeningitis—were recorded [12]. Furthermore, during several months of follow-up at the rheumatology outpatient clinic no clinical features suggestive of systemic disease were revealed.

#### 3.1.2. IgG4-Related Disease

Recent studies have drawn particular attention to IgG4-related disease (IgG4-RD), as dural thickening has increasingly been recognized as one of its possible clinical manifestations—this may suggest that cases previously considered idiopathic could, in fact, have a secondary etiology [14]. Investigators emphasize that symptoms reported by patients with IgG4-RD do not differ significantly from those seen in other forms of hypertrophic pachymeningitis [14] and the diagnosis should be supported by clinical involvement of other organs (e.g., orbits, lungs or retroperitoneum). The 2019 ACR/EULAR Classification Criteria for IgG4-RD are used to confirm or exclude this diagnosis [15,16,17]. Entry criteria include clinical or radiological involvement of a typically affected organ (e.g., pancreas, salivary glands, bile ducts, orbits, kidneys, lungs, retroperitoneum or thyroid) or histopathologic evidence of an inflammatory process with a lymphoplasmacytic infiltrate in any of these organs. In the presented patient, one of the affected organs was the thyroid gland—however, histopathologic examination revealed no features of IgG4-RD, allowing this diagnosis to be less likely. Moreover, imaging studies did not reveal other abnormalities suggestive of this disorder. Normal serum levels of IgG subclasses further argued against this diagnosis. However, it should be noted that IgG4-RD diagnostics have important limitations and complete exclusion of this disease without a dural biopsy is considered impossible [14]. Although biopsy remains the gold standard for diagnosing dural hypertrophy, its indications are not clearly defined due to limited evidence in the literature [18]. Should symptoms suggesting involvement of other organs appear, reassessment for IgG4-RD would be indicated, with particular emphasis on histopathologic evaluation.

#### 3.1.3. Infectious Causes

During the initial hospitalization, diagnostic tests to rule out common infectious causes of dural thickening were also performed. Literature reports highlight infectious agents—especially *Mycobacterium tuberculosis*, *Treponema pallidum* and *Borrelia burgdorferi*—as well as fungal infections [7,12]. Two lumbar punctures were performed and cerebrospinal fluid analyses showed no evidence of central nervous system infection. Both FTA and VDRL tests for syphilis were negative, and the absence of clinical manifestations, negative patient history, and non-progressive course of symptoms argued against this diagnosis [19].

As part of the differential diagnosis, neuroborreliosis was also considered. The patient lived in an endemic area but denied a history of erythema migrans, and serologic tests (IgM and IgG antibodies determined by ELISA and confirmed by Western blot) were negative, effectively excluding *Borrelia burgdorferi* infection as a potential cause of the dural changes [20]. Tuberculous meningitis may also lead to pachymeningitis; however, CSF analyses did not detect *Mycobacterium tuberculosis* and the Quantiferon test was negative.

#### 3.1.4. Neoplastic and Paraneoplastic Diseases

IHPM is not a common manifestation of metastatic disease [21]; however, a neoplastic cause should be carefully excluded before establishing the diagnosis, particularly given the presence of a proliferative process in this patient. The differential diagnosis included neoplastic diseases of the meninges—such as carcinomatosis, lymphoma, en plaque meningioma, melanoma [3], metastatic lesions and paraneoplastic syndromes [22].

Neoplastic processes may cause dural thickening. An association with thyroid cancer is poorly documented, however there is a published case of a patient with idiopathic hypertrophic pachymeningitis in whom metastatic papillary thyroid carcinoma was later diagnosed. Importantly, the neoplastic disease was identified three years after clinical improvement and partial radiological regression of dural changes, suggesting the coexistence of two distinct pathological entities rather than a secondary meningeal involvement [1].

Given the patient’s oncological history (microcarcinoma papillare confirmed on histopathological examination), a paraneoplastic etiology was also considered, potentially related to an autoimmune response triggered by tumor-associated antigens. However, additional test results argued against this hypothesis: CSF analyses revealed no onconeural antibodies or other abnormalities, while whole-body iodine-131 scintigraphy and repeated neuroimaging studies showed no evidence of disease recurrence or metastasis. Moreover, successive neuroimaging consistently demonstrated a diffuse and stable dural thickening rather than a focal lesion, further weakening the suspicion of a neoplastic process [23].

#### 3.1.5. Other Causes

Despite treatment, follow-up examinations revealed persistently elevated anti-TPO and anti-Tg antibody levels. Consequently, Hashimoto’s encephalopathy was considered in the differential diagnosis; however, this disorder typically presents with recurrent neuropsychiatric symptoms, including altered mental status, hallucinations, delusions, or seizures [24]—none of which were observed in the present case. Hashimoto’s encephalopathy is a rare autoimmune disorder (2 per 100,000 population), with anti-TPO antibodies detected in up to 100% of patients and anti-Tg antibodies in approximately 48%, though antibody titers do not correlate with disease severity [24]. The condition predominantly affects women. Most reported cases show no abnormalities on MRI, while described findings may include ischemic changes, demyelination, edema, atrophy, or signal alterations in the hippocampus or temporal lobes. Moreover, EEG usually includes nonspecific abnormalities [24], whereas the EEG in this patient was normal. These findings, however, may still suggest a possible autoimmune background of the symptoms, however thyroid autoantibodies themselves are not considered directly pathogenic in Hashimoto’s encephalopathy [24]. In the present case, the persistent elevation of anti-Tg titers may be interpreted as a marker of ongoing immune dysregulation.

Some literature suggests that Hashimoto’s disease may cause dural thickening [9]; however, no primary sources substantiate this claim. Clinical cases have been reported describing the coexistence of Hashimoto’s thyroiditis and Tolosa-Hunt syndrome, in which localized dural thickening has also been observed [25,26]; nonetheless, the character of the patient’s headache was not consistent with the ophthalmoplegic pain typical of Tolosa–Hunt syndrome.

Dural thickening may also be associated with CSF hypotension [22]. This diagnosis was considered because neuroimaging revealed downward displacement of the cerebellar tonsils into the spinal canal. However, lumbar puncture performed in May 2025 showed a CSF opening pressure of 80 mm H_2_O, whereas diagnostic criteria define intracranial hypotension as pressure < 60 mm H_2_O [22,27]. The absence of typical orthostatic symptoms further argues against this diagnosis.

#### 3.1.6. Differential Diagnosis Conclusions

Exclusion of the most common causes of dural thickening in this patient supports a diagnosis of idiopathic hypertrophic pachymeningitis, although an autoimmune mechanism appears probable. The persistently elevated anti-TPO and anti-Tg antibody levels, even several months after total thyroidectomy, may indicate the presence of an ongoing autoimmune inflammatory process. Importantly, persistently elevated anti-Tg antibodies are frequently observed after thyroidectomy in patients with Hashimoto’s thyroiditis or differentiated thyroid carcinoma and are not considered disease-specific nor directly pathogenic. Therefore, in the present case, the persistence of anti-Tg antibodies is interpreted as a nonspecific marker of ongoing immune dysregulation rather than a causal factor nor disease-specific autoimmune inflammatory process. A causal link may exist between an underlying immune dysregulation leading to the development of Hashimoto’s disease, complicated by papillary thyroid carcinoma and a subsequent autoimmune inflammatory reaction involving the dura mater, manifesting radiologically as dural hypertrophy and clinically significant. Importantly, elevated anti -Tg levels were already present at the initial neurological evaluation in September 2022, more than one year before the oncological diagnosis (Table 4). Following total thyroidectomy, anti-Tg concentrations remained persistently elevated, while repeated oncological follow-ups revealed no biochemical, imaging, or clinical evidence of residual, recurrent, or metastatic thyroid cancer.

### 3.2. Treatment

Given the rarity of the condition, its heterogeneous etiology, and variable clinical presentation, there are no established treatment guidelines for hypertrophic pachymeningitis, including its idiopathic form. Case analyses published to date suggest that oral corticosteroid therapy is effective in most patients during the early stages of the disease; however, tapering of steroid doses frequently leads to symptom recurrence, which typically resolves after re-escalation of corticosteroid therapy [1,28], which was also observed in this patient.

#### Steroid-Sparing Treatment Methods

Bosman et al. described a case of IHPM presenting with recurrent headaches, in which methotrexate was successfully introduced following the development of steroid resistance [29]. Another report presented three cases in which the addition of azathioprine after corticosteroid induction resulted in both clinical and radiological improvement [30]. Zhuoyou described a patient with idiopathic hypertrophic pachymeningitis successfully treated with intravenous cyclophosphamide infusion [31]. Other studies have also reported favorable outcomes of recurrent IHPM treated with methotrexate [32]. These observations suggest that alternative immunosuppressive strategies should be considered to minimize prolonged corticosteroid exposure and its associated adverse effects. However, this approach requires further investigation, as some previously reported cases may have involved secondary rather than idiopathic pachymeningitis.

An experimental study performed in 2019 has emphasized the need to clarify the underlying mechanisms of dural hypertrophy. In an animal model, the TGF-β1/SMAD2/SMAD3 signaling pathway was identified as a potentially key pathogenic mechanism in dural thickening, indicating possible avenues for future research and the development of targeted therapies [33].

In the presented case, the decision was made to refrain from steroid-sparing immunosuppressive therapy. This decision was based on the relatively mild disease course, with non-disabling symptoms that remained stable over several months of follow-up. The patient achieved symptomatic remission with corticosteroid therapy, which was well tolerated and not associated with significant adverse effects. Considering these factors, as well as the patient’s advanced age, the potential risks of escalating immunosuppressive treatment were deemed to outweigh the possible benefits. Therefore, a strategy of close clinical observation was adopted, with the option of reintroducing methylprednisolone in the event of symptom recurrence.

## 4. Conclusions

Hypertrophic pachymeningitis may remain an underrecognized condition due to the heterogeneity of its clinical presentation and etiology. Current evidence suggests that autoimmune mechanisms are a frequent underlying cause; however, large-scale studies investigating the relationship between immune-mediated disorders and neuroimaging abnormalities are lacking, making it difficult to clearly identify the pathophysiological basis of the disease. Some reports indicate that radiological regression on MRI occurs primarily in secondary forms of hypertrophic pachymeningitis, whereas in idiopathic cases radiological improvement tends to be limited, even when clinical remission is achieved [18].

Furthermore, standardized diagnostic and therapeutic protocols for pachymeningitis have not been established. The available literature remains fragmented, and reports on treatment strategies are inconsistent.

In the presented case, diffuse pachymeningitis persisted on neuroimaging, accompanied by elevated anti-Tg antibody titers despite the absence of imaging or clinical evidence of oncologic recurrence during post-thyroidectomy follow-up. Importantly, anti-Tg antibodies were elevated prior to the diagnosis of papillary thyroid microcarcinoma and remained persistently after total thyroidectomy, which was not accompanied by neoplastic disease activity. The patient exhibited corticosteroid-responsive symptoms without significant radiological improvement. Although the causal relationship cannot be established in a single case report, this observation should be regarded as hypothesis-generating and highlights the importance of systematically assessing autoimmune processes—including thyroid autoimmunity—in the differential diagnosis of IHPM.

## Figures and Tables

**Table 1:** Pathogenetic factors associated with IHPM [2,7,8,9,10,11].

Potential Pathogenetic Factors	Exemplary Diseases
Systemic diseases	Sarcoidosis, granulomatosis with polyangiitis, Sjögren’s syndrome, rheumatoid arthritis, IgG4-related disease, systemic lupus erythematosus, polyarteritis nodosa
Infectious	Tuberculosis, syphilis (especially neurosyphilis), Lyme disease, fungal infections
Neoplastic	Metastatic carcinoma to the dura mater, lymphoma, meningioma, reactive dural changes secondary to skull tumors
Other	Compensatory meningeal interstitial edema in response to decreased cerebrospinal fluid volume, mucopolysaccharidosis, intrathecal drug administration

Abbreviations: IHPM—idiopathic hypertrophic pachymeningitis.

**Table 2:** Methylprednisolone dose tapering schedule during the initial phase of therapy.

Treatment Phase	Methylprednisolone Dosage
Initial dose	64 mg, once daily in the morning (1.03 mg/kg)
After 7 days	32 mg, once daily in the morning (0.52 mg/kg)
After 14 days	16 mg, once daily in the morning (0.26 mg/kg)
After 3 months	8 mg, once daily in the morning (0.13 mg/kg)
After 5 months	4 mg, once daily in the morning * (0.06 mg/kg)
After 13 months	2 mg, once daily in the morning (0.03 mg/kg)
After 16 months	Discontinuation of corticosteroid therapy

* The dose reduction was considered ineffective; therefore, returning to the 8 mg dose for three consecutive months was recommended.

**Table 3:** Clinical course and treatment during follow-up visits.

Date of Visit	Reported Symptoms	Neurological Examination Findings	Recommended Treatment
January 2022	Vertigo with two episodes of vomiting	No abnormalities	Pentoxifylline 400 mg (2 times a day), thiethylperazine 6.5 mg (2 times a day)
September 2022	Vertigo during sudden positional changes, transient binocular diplopia	Mild dysmetria of the lower limbs, mild balance disturbances	No recommendations regarding steroid therapy
October 2022	Vertigo	No abnormalities	Methylprednisolone 64 mg for 1 week, then 32 mg for 1 week, followed by 16 mg
November 2022	Vertigo of lesser intensity	No abnormalities	Continuation of steroid therapy
January 2023	Memory disturbances	Mild balance disturbances	Reduction in methylprednisolone dose to 8 mg
May 2023	Vertigo, memory disturbances	Left limbs ataxia	Continuation of steroid therapy
June 2023	Unsteadiness, memory disturbances	No abnormalities	Reduction in methylprednisolone dose to 4 mg
July 2023	Vertigo	No abnormalities	Continuation of steroid therapy
September 2023	Headache, mild vertigo	No abnormalities	Continuation of steroid therapy
November 2023	Mild vertigo, mild balance disturbances	Left limbs ataxia, mild balance disturbances	Reduction in methylprednisolone dose to 2 mg
February 2024	No complaints	No abnormalities	Discontinuation of steroid therapy
April 2024	Mild balance disturbances	No abnormalities	No recommendations regarding steroid therapy
August 2024	Headache, nausea, vomiting	Mild balance disturbances	Methylprednisolone 16 mg
October 2024	Positional vertigo	Mild balance disturbances	Methylprednisolone 8 mg with gradual dose tapering
February 2025	Positional vertigo	Mild balance disturbances	In case of balance disturbances, consider initiation of methylprednisolone 16 mg
May 2025	Mild positional vertigo, occasional headache	Mild balance disturbances	No recommendations regarding steroid therapy
August 2025	Vertigo	Left limbs ataxia, gait disturbances, positive Romberg test	Methylprednisolone 16 mg with gradual dose tapering by half each week until discontinuation
October 2025	Mild balance disturbances, paroxysmal vertigo	Mild balance disturbances, limbs ataxia	Discontinuation of steroid therapy

**Table 4:** Changes in anti-TPO and anti-Tg antibody levels during follow-up.

Date	Anti-TPO (IU/mL)	Anti-Tg (IU/mL)
September 2022	451	748.31
* December 2023	-	-
February 2024	-	1130
April 2024	-	1000
August 2024	-	1039
November 2024	-	974
February 2025	78.6	1022
May 2025	69.7	1159

The table presents the dynamics of thyroid autoantibody (anti-TPO and anti-Tg) levels during the follow-up period. Following substernal thyroidectomy in 2023, persistently elevated anti-Tg titers were observed, accompanied by a gradual decline in anti-TPO concentrations. * Date the thyroidectomy was performed. Abbreviations: anti-TPO—anti-thyroid peroxidase antibodies, anti-Tg—anti-thyroglobulin antibodies, “-”—test not performed.

## Data Availability

The data presented in this study are available on request from the corresponding author due to privacy and ethical considerations related to patient confidentiality.

## Abbreviations

IHPM	Idiopathic hypertrophic pachymeningitis
Anti-Tg	Anti-thyroglobulin
CT	Computed tomography
MRI	Magnetic resonance imaging
TSH	thyroid-stimulating hormone
Anti-TPO	Anti-thyroid peroxidase
CSF	Cerebrospinal fluid
EEG	Electroencephalography
IgG4-RD	IgG4-related disease
